# Removal of the Gasserian Ganglion

**Published:** 1893-05

**Authors:** Edmund Andrews

**Affiliations:** Chicago, Ill.


### Removal of the Gasserian Ganglion.

BY EDMUND ANDREWS, M D., OF CHICAGO, ILL.

A Clinical Lecture Delivered at Mercy Hospital.

On Saturday, January 7, Dr. Edmund Andrews performed for the fourth time, at Mercy Hospital, Chicago, his operation for the removal of the Gasserian ganglion. The patient was a woman and furnished typical indications for the operation, having suffered for years with a tic douleureux of the right side. She had previously undergone two operations, she said, presumably section and avulsion of the nerve, with the usual result, viz.: a return of the agonizing pains in the course of about two months. When Dr. Andrews first conceived the idea of such an operation, he had satisfied himself that these obstinate trigeminal neuralgias usually have their origin in defective teeth which give rise to a neuritis involving first the inferior dental nerve and gradually progressing until the entire inferior maxillary branch of the fifth nerve is involved, that it ascends as far as the semilunar ganglion and stops there, not being transmitted beyond to the brain because of the difference in structure between the proximal and distal portions of the fifth nerve. His logical conclusion was that if the removal of the ganglion could be effected, the intolerable suffering of the patient would be relieved. After a great deal of experimentation upon the cadaver, six different methods of procedure were devised for the accomplishment of the end in view, one of which Dr. Andrews now regards as the best and will be herein briefly described as the writer witnessed it last Saturday.

The field of operations was rendered as aseptic as possible, the right side of the scalp being shaved and the utmost pains being taken to guard against infection. Beginning near the external angular process a curved incision was made extending horizontally above the zygomatic arch, curving down in front of the ear and terminating near the angle of the jaw, care being taken to avoid the pes anserinus. The flap thus made was turned down and stitched to the ala of the nose; the zygomatic arch was sawed through at each extremity and reflected down carrying with it the attached masseter muscle; the coronoid process of the lower jaw was then sawed off and turned up on to the temple, carrying with it the attachment of the temporal muscle, and was stitched to the scalp. Now some fat and connective tissue was exposed containing in it the internal maxillary artery, which was tied. Persistent oozing hemorrhage had compromised each step of the operation and was particularly troublesome at this stage; the blood would well up into the wound almost as fast as an assistant could sponge it away, and owing to this masking of the parts involved, the operator had to literally feel his way in the dark. Search was now made for the inferior dental nerve which should serve as a guide to the foramen ovale, but it was not found, owing no doubt to the fact that it had been entirely torn away at one of the former operations. Therefore the finger of the operator felt for the posterior sharp edge of the external pterygoid plate of the sphenoid bone and passed it up to its base, near which the foramen is situated, and through which a probe was passed, the ganglion being transfixed thereby. Just external to the foramen ovale is a triangular free space on the under surface of the base of the skull, and here a specially devised trephine with a long shank and a long center pin was applied, and the button of bone removed. The narrow isthmus of bone intervening between the trephine hole and the foramen ovale was bitten away with a pair of rongeur forceps and through the large opening thus made, a surgical spoon was passed and the ganglion scraped away.

In closing up the wound Dr. Andrews advocates wiring together the bones sawed through, but in this particular case it became necessary to dispense with this procedure, owing to the manifestation by the patient of symptoms of collapse—due chiefly to the hemorrhage, partly also to shock—from which however she rallied nicely after being put to bed, receiving hypodermic injections of digitalis and strychnine and the application of heat.

Time required for the operation was about two hours. To the bystander it seemed a formidable one—as it was a tedious one to the operator. But it must be remembered that the patients suffering with this trouble will invite almost any conditions whatever that will offer them escape, and always say that if the operation won't bring them relief, they prefer not to awake from the anæsthetic.

